# Assessing the Impact of the Updated 2021 European Respiratory Society/American Thoracic Society Criteria on Bronchodilator Responsiveness in Asthma

**DOI:** 10.7759/cureus.66844

**Published:** 2024-08-14

**Authors:** Nina Trepić, Marko Nemet, Marija Vukoja

**Affiliations:** 1 Internal Medicine, Faculty of Medicine, University of Novi Sad, Novi Sad, SRB; 2 Pulmonology, Institute for Pulmonary Diseases of Vojvodina, Sremska Kamenica, SRB

**Keywords:** respiratory function tests, clinical guidelines, bronchodilator effect, spirometry, diagnosis, asthma

## Abstract

Introduction

The European Respiratory Society/American Thoracic Society (ERS/ATS) Task Force released new technical standards on spirometry interpretation in 2021. Our study compares bronchodilator responsiveness (BDR) in asthma, evaluating the impact of the 2005 and 2021 ERS/ATS criteria and the influence of predictive equations.

Methods

A retrospective cohort study of adult patients with asthma was referred to spirometry with a BDR test at the Institute for Pulmonary Diseases of Vojvodina, Sremska Kamenica, Serbia. The study included adult patients with asthma who underwent BDR testing in the Department of Respiratory Pathophysiology at the institute and had available data on height, gender, age, race, and eosinophil count.

Results

Among 197 patients, 69 were men (35.0%), the median age was 47 years (interquartile range (IQR) 38-60), and a positive BDR test occurred in 50 (25.38%) according to the 2005 criteria and 42 (21.32%) according to 2021 criteria when using the Global Lung Initiative (GLI) reference equations. Strong agreement was observed between the ERS/ATS 2005 and 2021 criteria (Cohen’s kappa index: 0.887, 95%, CI 0.810 to 0.963). Similar results were found with the Third National Health and Nutrition Examination Survey (NHANES III) and the European Community of Coal and Steel (ECCS) predictive equations. Positive BDR tests were common in patients with moderately severe and severe forced expiratory volume in one second (FEV1) impairment and were not associated with eosinophil count or total serum immunoglobulin E (IgE) levels.

Conclusion

The introduction of the 2021 ERS/ATS criteria did not significantly alter the classification of BDR in the majority of asthma patients, ensuring diagnostic stability. Whichever criterion was used, positive BDR correlated with the extent of FEV1 impairment, but not asthma biomarkers.

## Introduction

Spirometry is widely used to assess pulmonary function for the screening and diagnosis of lung diseases [1]. Bronchodilator responsiveness testing (BDR) using short-acting bronchodilators (BDs) is an important part of the patient evaluation. This test allows for the determination of the degree of volume and airflow improvement following the administration of BDs and is used to assess airway responsiveness, which is a hallmark of asthma [2].

According to the European Respiratory Society/ American Thoracic Society (ERS/ATS) International Joint Task Force interpretation statement, issued in 2005, a combination of absolute and relative changes in forced expiratory volume in one second (FEV1) and forced vital capacity (FVC) from baseline is recommended to determine BDR. Specifically, an increase of 200 ml or more and 12% or more in FEV1 and/or FVC is considered evidence of BDR [3]. The 2005 BDR criteria are also recommended by the Global Initiative for Asthma (GINA) [4] and the ERS Guidelines for the Diagnosis of Asthma in Adults [3] as part of the diagnostic criteria for asthma. However, it is important to note that BDR can vary over time due to several factors. Variability in BDR can be influenced by the patient's baseline lung function, severity of the disease, and current medication use. In addition, environmental factors and seasonal and diurnal variations can impact the degree of airway responsiveness [5,6].

To ensure consistency and accuracy in spirometry, the ERS/ATS International Joint Task Force updated their 2005 recommendations and released new technical standards for interpreting routine lung function tests in 2021 [1]. The updated interpretation of BDR testing was necessary because absolute and relative changes in FEV1 and FVC were found to be closely related to baseline lung function level and, therefore, biased toward height, age, and gender [7]. Reporting BDR as a percentage change compared to the individual's expected value may aid in reducing gender and size bias [7]. As a consequence, according to the 2021 ERS/ATS interpretation techniques, a positive response is defined as an increase of more than 10% from the predicted value for FEV1 or FVC [1]. The interpretation of the 2021 BDR is therefore dependent on the use of relevant FEV1 and FVC predicted values. These values should be calculated using population-based reference equations derived from large and representative samples of healthy individuals with similar anthropometric characteristics as the subject, taking into account the dependence of lung function on age [3].

The Global Lung Initiative (GLI) 2012 reference equations are the most accurate spirometric prediction equations for the age range of three to 95 years and are endorsed by major international respiratory societies [8]. However, different parts of the world may still use different reference equations. For instance, until recently, the Third National Health and Nutrition Examination Survey (NHANES III) equations were recommended in the United States [9], and European Community for Coal and Steel (ECCS) equations were utilized in many European countries [10]. Despite their reliability, NHANES III equations are limited to Caucasians, African Americans, and Hispanics aged eight to 80 years. The ECCS values were developed based on White Caucasian males between 18 and 70 years of age, working in steelworks and coal mines, and the values for women were estimated as 80% of those for males [8].

The primary aim of our study was to compare the ERS/ATS 2021 criteria for BDR testing with the widely adopted 2005 criteria. In addition, our study aims to investigate the influence of different predictive equations on the assessment of BDR according to the new criteria and examine clinical characteristics associated with BDR, using both the 2005 and 2021 criteria. Understanding the differences in BDR criteria could enhance diagnostic precision, guiding practitioners in optimizing patient care and treatment strategies. 

The preliminary results of the study were previously presented as a meeting abstract at the 2023 ERS International Congress on September 11, 2023 [11].

## Materials and methods

Study design

This retrospective observational study took place at the Institute for Pulmonary Diseases of Vojvodina, Sremska Kamenica, Republic of Serbia. The Institute for Pulmonary Diseases of Vojvodina serves as a tertiary healthcare facility in the Province of Vojvodina, Republic of Serbia. Complying with the Health Insurance Portability and Accountability Act (HIPAA) regulations and ensuring subject confidentiality, all collected data were de-identified, eliminating the requirement for written informed consent. The study received approval from the Ethics Committee of the Institute for Pulmonary Diseases of Vojvodina, with the assigned approval number 16-IX/8, ensuring adherence to ethical standards and protocols.

Data resource and study population

The study is based on data collected from the database of the information system of the Institute for Pulmonary Diseases of Vojvodina. The study included adult patients with asthma who underwent BDR testing in the Department of Respiratory Pathophysiology at the Institute from January 1, 2018, to December 31, 2022. Patients with asthma were identified based on the International Statistical Classification of Diseases 10th revision (ICD-10) codes (J45.0-J45.9). Patients were selected from the database if they met the following inclusion criteria: ICD-10 code for asthma, BDR test, and eosinophil count. Exclusion criteria were applied to filter out incomplete data on key variables such as age, gender, and height (necessary to calculate different sets of predictive equations) and those with co-existing respiratory conditions that might influence study outcomes (e.g., chronic obstructive pulmonary disease (COPD) and bronchiectasis). All retrieved records underwent manual review to ensure accuracy. Initially, 203 patient reports were extracted from the database. After applying exclusion criteria, a total of 197 patient reports were included in the final analysis. For patients who performed BDR testing more than once, only the initial report was selected. The sample size for this research was determined based on the availability of patient information through electronic medical records, rather than through a formal calculation prior to the study’s initiation. This approach enabled the utilization of readily accessible data from a select group of patients.

Spirometry tests

Spirometry tests were performed by trained technicians according to the ERS/ATS guidelines [1,3]. The BDR test was performed by using four separate actuations of 100 mcg of salbutamol (total 400 mcg) with a metered-dose inhaler through a spacer, and spirometry was repeated after 15 to 30 minutes [3].

Lung function indices

Lung function indices were recorded before and after BD administration. The standard indices included FEV1, FVC, and FEV1/FVC. These parameters are presented as absolute values and percent predicted (%). Positive and negative BDR test results were defined based on the 2005 (BDR 2005) and 2021 (BDR 2021) ERS/ATS criteria and were classified as BDR+ and BDR-, respectively. Predictive values were derived from the GLI-2012, NHANES III, and ECCS equations. The severity of FEV1 impairment was assessed based on the z-scores and classified as follows: normal if FEV1 z-score ≤ -1.64, mild if FEV1 -2 ≤ z-score ≥ -1.64, moderate if -2.5 ≤ FEV1 z-score < -2, moderately severe if -3 ≤ FEV1 z-score < -2.5, severe if ≤ -4 FEV1 z-score < -3, and very severe if FEV1 z-score < -4 [12].

Statistical analysis

A descriptive statistical analysis of relevant data was performed. Quantitative variables are presented as the mean and standard deviation or median and interquartile range (IQR) depending on the distribution for continuous variables and frequency with the percentage for categorical variables. The comparison between BDR 2005 and BDR 2021 was assessed through kappa agreement statistics, where a value of 0-0.20 indicates none, 0.21-0.39 minimal, 0.40- 0.59 weak, 0.60-0.79 moderate, 0.80-0.90 strong, and above 90 almost-perfect agreement [13]. The comparison between baseline characteristics, lung function indices, and markers of T helper 2 cell (Th2) inflammation (eosinophil count and serum immunoglobulin E (IgE) levels) between BDR+ and BDR- subjects using the 2005 and 2021 criteria was performed by Kruskal-Wallis test for not normally distributed continuous variables, T-test for two independent means for normally distributed continuous variables, and Chi-square test for categorical variables. The p-values <0.05 were considered statistically significant. The statistical analysis was conducted using the R programming language (R Core Team (2021), R: A language and environment for statistical computing, R Foundation for Statistical Computing, Vienna, Austria) and the RStudio software program, version 2023.03.1+446 (RStudio Team (2020), RStudio: Integrated Development for R, RStudio, PBC, Boston, MA).

## Results

Baseline characteristics

A total of 197 patient reports that met the inclusion criteria were extracted from the database and were included in the final analysis. There were 69 men (35.0%), and the median age was 47 years (IQR 38-60). The baseline characteristics of patients are presented in Table 1. 

**Table 1:** Patients' baseline characteristics Abbreviations: IQR: interquartile range, IU: international units

Parameter	Value
Age (years), median (IQR)	47.0 (38.0-60.0)
Male gender, n (%)	69 (35.00)
FEV1 (L), median (IQR)	2.48 (1.80-3.28)
FEV1%, median (IQR)	80.72 (62.55-95.19)
FVC (L), median (IQR)	3.65 (3.01-4.44)
FVC%, median (IQR)	92.38 (82.06-103.10)
FEV1/FVC, median (IQR)	0.68 (0.58-0.79)
Eosinophils (x10^9^/L), median (IQR)	0.25 (0.13-0.43)
IgE (IU/mL) ( n=117), median (IQR)	97.28 (34.17-304.75)

**Figure 1:**
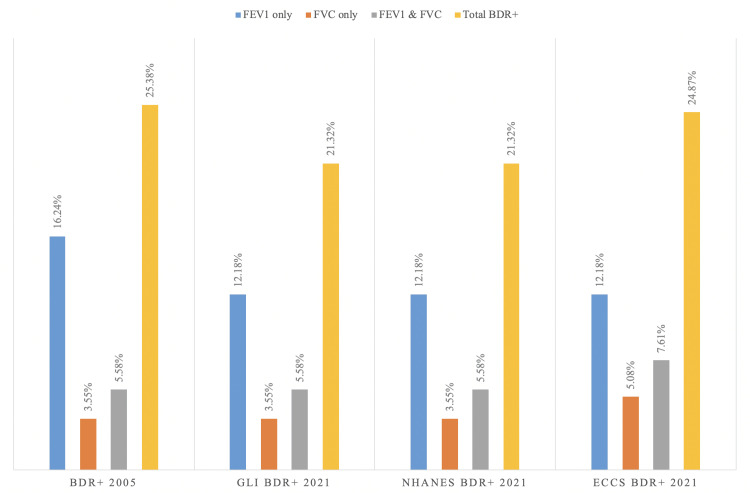
Percentage of BDR+ test using 2005 and 2021 ERS/ATS criteria Abbreviations: BDR: bronchodilator responsiveness, ERS/ATS: European Respiratory Society/American Thoracic Society

A positive BDR was present in 50 patients (25.38%) according to the ERS/ATS 2005 criteria and 42 patients (21.32%) according to the ERS/ATS 2021 criteria using GLI reference equations (p = 0.34). A positive test result was based on FEV1 in 32 (16.24%), FVC in seven (3.55%), and on both FEV1 and FVC in 11 (5.58%) of the patients according to the 2005 criteria, while according to the 2021 criteria, positivity was based on FEV1 in 24 (12.18%), FVC in seven (3.55%), and on both FEV1 and FVC in 11 (5.58%) patients. The results are displayed in Figure 1.

Overall, there was strong agreement between the ERS/ATS 2005 and 2021 criteria based on the GLI reference equations (Cohen’s kappa index: 0.887, 95%, CI 0.810 to 0.963). This agreement was almost perfect in females (kappa 0.935, 95%, CI 0.863 to 1.00). In males, the agreement was moderate (kappa 0.794, 95%, CI 0.623 to 0.964), as seen in Table 2.

**Table 2:** Agreement between ERS/ATS 2005 and 2021 BDR criteria based on GLI reference equations Data are shown as n (%). Abbreviations: BDR+: positive bronchodilator responsiveness test, BDR-: negative bronchodilator responsiveness test, CI: confidence interval

BDR 2021	BDR+ 2005	BDR- 2005	Cohen’s kappa index (95% CI)
BDR+2021	42 (21.32)	0 (0.00)	0.887 (0.810-0.963)
BDR- 2021	8 (4.06)	147 (74.62)	
Male			
BDR+2021	13 (18.84)	0 (0.00)	0.794 (0.623-0.964)
BDR- 2021	5 (7.25)	51 (73.91)	
Female			
BDR+2021	29 (22.66)	0 (0.00)	0.935 (0.863-1.00)
BDR- 2021	3 (2.34)	96 (75.00)	

Differences between the percentages of the 2005-BDR+ and 2021-BDR+ using NHANES III and ECCS criteria

Using the NHANES criteria for the determination of predictive values, a positive BDR was present in 42 patients (21.32%) according to the ERS/ATS 2021 criteria. A positive BDR was based on FEV1 in 24 (12.18%), FVC in seven (3.55%), and both FEV1 and FVC in 11 (5.58%) patients (Figure 1). Overall, there was a strong agreement between the ERS/ATS 2005 and 2021 criteria (kappa index: 0. 859, 95%, CI 0.774 to 0.943). This agreement was strong in females (kappa 0.892, 95%, CI 0.800 to 0.985) and moderate in males (kappa 0.794, 95%, CI 0.623 to 0.964) as shown in Table 3.

**Table 3:** Agreement between the ERS/ATS 2005 and 2021 BDR criteria based on the NHANES III reference equations Data is shown as n (%). Abbreviations: ERS/ATS: European Respiratory Society/American Thoracic Society, BDR: bronchodilator responsiveness, NHANES III: Third National Health and Nutrition Examination Survey

BDR 2021	BDR+ 2005	BDR- 2005	Cohen’s Kappa index (95% CI)
BDR+ 2021	41 (20.81)	1 (0.51)	0.859 (0.774 - 0.943)
BDR- 2021	9 (4.57)	146 (74.11)	
Male			
BDR+ 2021	13 (18.84)	0 (0.00)	0.794 (0.623 - 0.964)
BDR- 2021	5 (7.25)	51 (73.91)	
Female			
BDR+ 2021	28 (21.88)	1 (0.78)	0.892 (0.800 - 0.985)
BDR- 2021	4 (3.12)	95 (74.22)	

Using the ECCS criteria for the determination of predictive values, a positive BDR was present in 49 patients (24.87%) according to the ERS/ATS 2021 criteria. A positive test result was based on FEV1 in 24 (12.18%), FVC in 10 (5.08%), and both FEV1 and FVC in 15 (7.61%) patients (Figure 1). Overall, there was a strong agreement between the ERS/ATS 2005 and 2021 criteria (kappa index: 0.852, 95%, CI 0.767 to 0.937). This agreement was similar in males (kappa 0.838, 95%, CI 0.686 to 0.990) and females (kappa 0.859, 95%, CI 0.757 to 0.960) as shown in Table 4.

**Table 4:** Agreement between the ERS/ATS 2005 and 2021 BDR criteria based on the ECCS reference equations Data is shown as n (%). Abbreviations: ERS/ATS: European Respiratory Society/American Thoracic Society, BDR: bronchodilator responsiveness, ECCS: European Community of Coal and Steel

BDR 2021	BDR+ 2005	BDR- 2005	Cohen’s kappa index (95% CI)
BDR+2021	44 (22.33)	5 (2.54)	0.852 (0.767-0.937)
BDR- 2021	6 (3.05)	142 (72.08)	
Male			
BDR+2021	14 (20.29)	0 (0.00)	0.838 (0.686-0.990)
BDR- 2021	4 (5.80)	51 (73.91)	
Female			
BDR+2021	30 (23.44)	5 (3.91)	0.859 (0.757-0.960)
BDR- 2021	2 (1.56)	91 (71.09)	

Differences between BDR- and BDR+ within the 2005 and 2021 criteria using the GLI equations

The differences in demographic characteristics, lung function, and markers of Th2 inflammation between positive and negative patients using the 2005 and 2021 criteria are presented in Table 5. 

**Table 5:** Differences in pulmonary function indices and asthma biomarkers between the 2005-BDR- and + and 2021-BDR- and + groups based on the GLI reference equations Data are shown as median with Interquartile range. Abbreviation: GLI: Global Lung Initiative

Parameter	2005 BDR- N=147	2005 BDR+ N=50	p value	2021 BDR- N=155	2021 BDR+ N=42	p-value
Male, n (%)	51 (34.69)	18 (36.0)	0.867	56 (36.13)	13 (30.95)	0.533
Age (years)	46 (36-57)	52.5 (40.5-63.5)	0.013	46 (36-57)	55 (41-64)	0.006
Height (cm)	167.00 (162.00-176.50)	166.00 (161.25-177.00)	0.641	167.00 (162.00-177.00)	166.00 (160.25-174.50)	0.528
BMI (kg/m2)	26.64 (23.69-31.19)	26.44 (23.70-28.99)	0.510	26.50 (23.52-30.96)	27.74 (24.00-29.72)	0.471
FEV1 (L)	2.80 (2.08-3.49)	1.87 (1.46-2.37)	<0.001	2.75 (2.06-3.46)	1.87 (1.48-2.33)	<0.001
FEV1%	87.14 (72.80-9.29)	60.68 (52.47-75.22)	<0.001	85.97 (69.31-97.90)	64.25 (53.79-77.72)	<0.001
FVC (L)	3.73 (3.15-4.46)	3.43 (2.69-4.25)	0.033	3.73 (3.15-4.47)	3.05 (2.63-4.14)	0.007
FVC%	93.59 (83.66-105.19)	89.66 (80.24-95.22)	0.009	92.90 (83.66-104.99)	88.10 (79.20-95.22)	0.010
FEV1/FVC	0.73 (0.64-0.82)	0.56 (0.51-0.65)	<0.001	0.72 (0.62-0.82)	0.58 (0.51-0.66)	<0.001
Eosinophils (x109/L)	0.24 (0.12-0.415)	0.27 (0.15-0.55)	0.260	0.24 (0.12-0.42)	0.27 (0.15-0.58)	0.662
IgE (IU/mL) ( n=117)	96.815 (44.76-285.4)	130.58 (17.82-394.71)	0.966	97.00 (44.50-320.12)	119.30 (16.61-294.25)	0.457

As shown in Table 5, the BDR-negative patients were younger than the BDR-positive patients while using both the 2005 and 2021 criteria. Furthermore, the values for FEV1, FEV1%, FVC, FVC%, and FEV1/FVC were lower in the groups with a positive BDR than in the BDR-negative groups. There were no differences between the groups regarding gender, height, BMI, and disease biomarkers (eosinophil count and IgE levels).

Trend in the proportion of BDR+ and the degree of FEV1 impairment

**Figure 2:**
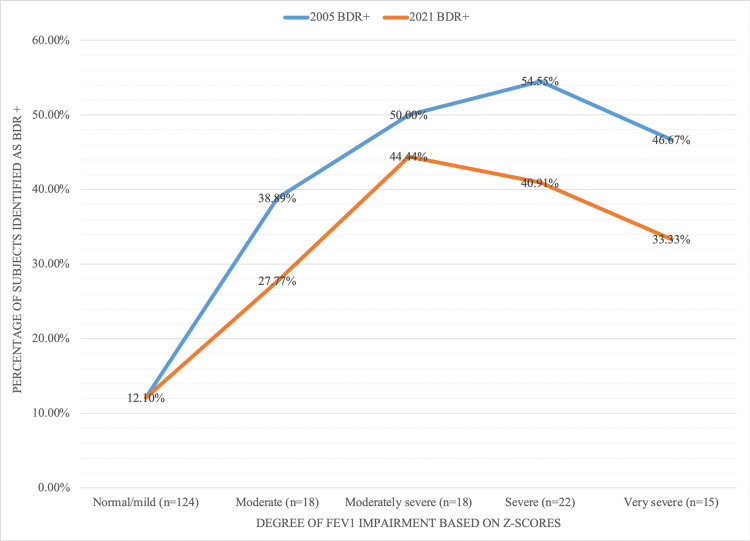
Trend in the proportion of BDR+ with the increased degree of FEV1 impairment in asthma Normal/mild if FEV1 z-score ≥ -2; moderate if -2.5 ≤ FEV1 z-score < -2; moderately severe if -3 ≤ FEV1 z-score < -2.5; severe if ≤ -4 FEV1 z-score < -3; very severe if FEV1 z-score < -4; n = total number of subjects in a specific FEV1 z-score category. Abbreviations: BDR+: positive bronchodilator responsiveness test, FEV1: forced expiratory volume

The association between the number of positive tests and the severity of FEV1 impairment showed an inverted U-shaped curve when using both the 2005 ERS/ATS criteria (p<0.001) and the 2021 ERS/ATS criteria (p<0.001), as shown in Figure 2. 

Both graphs exhibited a low percentage of BDR+ tests in patients with normal FEV1 or mild FEV1 impairment, and the percentage of a positive BDR increased in patients with moderate and severe FEV1 impairment and declined in patients with very severe reduction of FEV1. 

## Discussion

The present study found a slight reduction in positive BDR when using the 2021 ERS/ATS criteria compared to the 2005 ERS/ATS criteria. However, the overall agreement was strong regardless of the predictive equation used. The BDR positivity was associated with the level of FEV1 impairment and age and was not associated with markers of Th2 inflammation when assessed by both the 2005 and 2021 criteria.

The choice of a threshold for a positive BDR in asthma is considered arbitrary since the reversibility of airway obstruction in response to BDs is a continuous variable [14]. For instance, FEV1 values could be used to differentiate patients based on survival advantage or improvement. A change in FEV1 of more than eight percent predicted has been linked to a survival benefit over non-responsive individuals [7], whereas a change in FEV1 value of more than four percent predicted has been utilized to distinguish patients considered improved based on peer reviews [15]. According to the 2021 guidelines, a change of >10% separates responders from the healthy population. Thus far, only a few studies evaluated the impact of newly established BDR criteria, with conflicting results. A recent large cohort study from China showed a significant reduction in the percentage of 2021-BDR+ tests compared to 2005-BDR+ in both, subjects with asthma and chronic obstructive pulmonary disease (COPD) [16]. By contrast, a study by Bhatt et al. reported an increase in the prevalence of BDR+ in subjects with COPD with the 2021-BDR+ criteria. However, in the same study, BDR positivity according to either the 2005 or 2021 criteria did not influence patients’ outcomes [17]. The differences in findings may be due to subject heterogeneity, including age, underlying disease, ethnicity, and sample size.

A study by Quanjer et al. showed an association of BDR with age, height, sex, and level of respiratory impairment in patients with obstructive disease [18]. This suggests that differences in pulmonary function caused by underlying respiratory conditions can lead to different spirometry outcomes in similar population samples. Regarding ethnicity, presumed biological differences in pulmonary function between populations may also manifest as differences in spirometry outcomes [3]. Moreover, larger sample sizes provide better insights into population-wide trends, while smaller samples may exhibit more variability.

In our study, there was strong agreement between the two sets of criteria, with almost-perfect agreement among females and moderate agreement among males. The observation that the shift from positive to negative BDR test results was primarily connected to men is consistent with the original result that the 2005 criteria were biased toward identifying men as positive more frequently than females [19]. According to Ward et al., the 2005 criteria favored those with a lower FEV1 at baseline with at least a 12% change relative to the starting value, while at least a 200 mL change favored males [7]. By contrast, a change in FEV1 as a percentage of predicted values avoids biases related to sex, weight, and height and is better at predicting survival [20].

There can be significant variations in the predicted values of FEV1 and FVC depending on the prediction equations used. As a result, the interpretation of the severity of airway obstruction may differ based on the choice of equations [21]. In our study, we utilized the GLI prediction equations for determining the reference values for BDR testing. Those equations are widely accepted as the suggested reference equation by numerous national respiratory associations [8]. To make our study more comparable, we also used the ECCS and NHANES III prediction equations to calculate reference values, since it could be important as the denominator for 2021 criteria. When using the NHANES III criteria, the obtained results were almost identical to those derived from the GLI predictive equations. These findings are consistent with previous studies, which demonstrated no significant variation in the restrictive pattern between the GLI and NHANES [22] and where the use of NHANES III predicted equations led to less misclassification of lung function impairment than ECCS [21]. The observed significant agreement between the GLI and NHANES predictive equations highlights the reliability of the ERS/ATS 2021 criteria and the possibility of their widespread clinical applicability in different countries of the world despite using different predictive equations.

In the last 20 years, the ECCS equations were the standard equations for the majority of European countries; nonetheless, these equations have significant drawbacks. The ECCS issued sets of reference values focused on males and included individuals working in coal mines and steel works, resulting in a non-representative reference population and predicted values that are regarded to be too low [23]. When using the ECCS for calculating reference values in the present study, there was an increase of seven (3.55%) in the number of patients with a BDR+ test result, but this did not significantly influence the results. It is important to point out that the patients, who were found to have a non-significant BDR according to 2005 criteria, but were later classified as BDR+ according to the 2021 criteria, were all females. A possible explanation for this finding may be that, regarding the new standards, there is no longer an absolute change criterion [19]. Although we observed a high level of concordance between the 2005 and 2021 criteria regardless of the predictive equation used, physicians should keep in mind that certain patients may still be reclassified with newly proposed criteria. This reclassification is due to the effects of gender, height, and initial spirometry values on BDR and is somewhat dependent on the choice of predictive equation.

Interestingly, BDR-negative patients were consistently younger than their BDR-positive patients, a trend observed under both the 2005 and 2021 criteria. One potential explanation for this age difference might be the natural aging process, as older individuals could experience age-related changes in lung function that impact the likelihood of demonstrating BDR [18]. Another plausible hypothesis could be related to variations in disease duration or severity, with younger patients potentially representing an earlier stage of disease progression.

In our study of predominantly mild asthma patients, most BDR+ were based on the FEV1 increase only. This could be due to the fact that FEV1 is predominantly influenced by airflow restrictions at high lung volumes, whereas FVC is mostly influenced by low lung volume [24,25]. When the pattern of BDR is examined in relation to alterations in both FEV1 and FVC, its clinical importance increases. For instance, a post-bronchodilator change in FEV1 only is more prevalent in mild and moderate stages of COPD, but not in severe. On the other hand, a change in FVC only is more prevalent in the advanced stages of COPD as it is related to the presence of air trapping and dynamic hyperinflation [22]. With the increase of FEV1 impairment, the percentage of BDR+ increased in the 2005 ERS/ATS and 2021 ERS/ATS, except in those with a very severe reduction in FEV1. The number of positive tests using the 2005 and 2021 criteria showed similar trends across disease severity with the largest discrepancy in patients with severe airflow limitation (reduction in number of positive tests by 14% when the 2021 criteria were used). Patients with lower FEV1 may fulfill the 2005 BDR percentage criterion despite being less likely to meet the absolute volume threshold [26]. This is because the absolute volume criterion is unaffected by the initial value, and expressing change as %baseline indicates that the worst initial values are associated with the highest reversibility. This effect is minimized by stating change as a percentage of predicted [18].

Although high eosinophil count and IgE levels suggest Th2 inflammation and are used to support the diagnosis of asthma [27], we found no association between these biomarkers and positive response using either criterion, which is in line with the previously published data that defined positivity based on the 2005 criteria [26]. The lack of association between BDR and markers of Th2 cell inflammation suggests potential limitations in using traditional biomarkers to predict or understand response patterns. However, these results should be interpreted with caution as there is a possibility that the current study was underpowered to detect any noticeable changes in disease biomarkers (eosinophil count and IgE levels).

Our study has several limitations. First, we mainly concentrated on the differences in baseline pulmonary indices and disease biomarkers but did not include indices related to clinical outcomes such as symptoms, general health status, exercise capacity, exacerbations, and mortality. To evaluate the prognosis and validity of the new criteria, it is of utmost importance to incorporate relevant clinical factors. Second, the presence of BDR has been shown to fluctuate over time [28,29], and the results from a single BDR test should be regarded with caution. Third, the relatively limited sample size decreased the statistical power while increasing the risk of type II error, thus potentially preventing the detection of all significant associations. Furthermore, official sample size calculations were not performed prior to the initiation of the study.

Despite its limitations, our study is one of the first studies that assessed the discrepancies between the old and new BDR criteria. Furthermore, it is currently the only study, to our knowledge, to have evaluated these criteria using and comparing the GLI, NHANES III, and ECCS reference equations. This enhances the generalizability of our findings and provides a comprehensive assessment of the different prediction equations that are commonly used worldwide. This is of particular importance since many countries have yet to transition to the GLI due to associated equipment costs. Therefore, our study’s use of multiple prediction equations is relevant and beneficial for clinical practice, especially in developing countries.

## Conclusions

Compared to the 2005 ATS/ERS BDR criteria, the use of the 2021 ATS/ERS BDR criteria, although more restrictive, did not cause a significant change in the percentage of positive tests in patients with asthma. The two criteria showed almost perfect agreement in women and moderate-to-strong agreement in men. The positive test was mostly found in patients with moderate-severe FEV1 impairment and was not associated with markers of Th2 inflammation regardless of which criteria were used. This consistency is particularly important for clinicians relying on BDR as a diagnostic criterion for asthma. However, clinicians should be aware of the impact of predictive reference equations on the interpretation of new BDR criteria and a possible misclassification using old BDR criteria in certain patients.
